# TNF-alpha inhibitors reduce the incidence of PsA in patients with psoriasis: a propensity score-matched cohort study

**DOI:** 10.1093/rheumatology/keaf364

**Published:** 2025-07-03

**Authors:** Stefano Piaserico, Matteo Megna, Federico Bardazzi, Michela Magnano, Giulia Giovanardi, Giulia Odorici, Clara De Simone, Roberta Ramonda, Andrea Conti, Dennis McGonagle

**Affiliations:** Unit of Dermatology, Department of Medicine, University of Padua, Padua, Italy; Department of Dermatology, University of Naples Federico II, Napoli, Italy; Dermatology Unit, IRCCS Azienda Ospedaliero-Universitaria di Bologna, Policlinico S.Orsola Malpighi, Bologna, Italy; Dermatology Unit, IRCCS Azienda Ospedaliero-Universitaria di Bologna, Policlinico S.Orsola Malpighi, Bologna, Italy; Department of Dermatology, Catholic University of the Sacred Heart, Policlinico Universitario “A.Gemelli”, IRCCS, Rome, Italy; Department of Medical Sciences, Section of Dermatology and Infectious Diseases, University of Ferrara, Ferrara, Italy; Department of Dermatology, Catholic University of the Sacred Heart, Policlinico Universitario “A.Gemelli”, IRCCS, Rome, Italy; Unit of Rheumatology, Department of Medicine, University of Padua, Padua, Italy; Dermatology Unit, Ospedale Infermi di Rimini, AUSL Romagna, Rimini, Italy; Leeds Institute of Rheumatic and Musculoskeletal Medicine, University of Leeds and NIHR Leeds Biomedical Research Centre, Leeds Teaching Hospitals Trust, Leeds, UK

**Keywords:** psoriasis, PsA, prevention, TNF inhibitors, narrow-band UVB phototherapy

## Abstract

**Objectives:**

Conflicting data exist on TNF inhibitors’ (TNFi) role in preventing PsA in psoriasis. Using propensity score matching, we compared PsA incidence in severe psoriasis patients treated with TNFi vs narrow-band ultraviolet B (nbUVB) phototherapy over a decade of follow-up.

**Methods:**

Consecutive adults with severe psoriasis prescribed TNFi or nbUVB phototherapy between September 2005 and September 2010 were enrolled. Of 946 patients, 497 received TNFi (median follow-up 9.6 ± 2.6 years) and 449 underwent nbUVB (9.4 ± 5.9 years). All had rheumatologist assessment before therapy and for PsA diagnosis. PS matching was adjusted for factors linked to PsA, including arthralgia, family history, BMI, PASI and psoriasis distribution, including nails.

**Results:**

After propensity score matching, the TNFi cohort contributed 2705.5 person-years of follow-up (mean 9.1 ± 2.9 years), and the nbUVB cohort contributed 2654.1 person-years (mean 8.9 ± 5.4 years). The PsA incidence rate per 100 patients was 1.18 (0.84–1.52) in the TNFi group and 2.48 (2.24–2.72) in the nbUVB group, yielding an incidence rate ratio of 2.1 (1.37–2.98, *P* = 0.0002). A time-dependent Cox model confirmed that TNFi treatment was associated with a significantly lower risk of PsA (HR = 0.32, *P* < 0.0001). Arthralgia (HR = 7.68, *P* < 0.0001), nail psoriasis (HR = 1.93, *P* = 0.0004) and higher PASI score (HR = 1.03 per point, *P* = 0.0096) were independent predictors of PsA.

**Conclusion:**

This PS-matched study shows a clear benefit of TNFi vs nbUVB in PsA reduction in severe psoriasis patients over nearly a decade of therapy.

Rheumatology key messages

- Conflicting reports exist on the impact of TNFi use in psoriasis and their ability to prevent PsA development.
- In this long-term propensity score-matched cohort study of 946 patients with severe psoriasis, TNFi use halved the risk of developing PsA compared to phototherapy treatment.
- The findings suggest that prolonged TNF inhibitors treatment plays a protective role in PsA development in severe psoriasis patients.

## Introduction

Psoriasis is a chronic immune-mediated skin disease affecting ∼2% of the general population [1]. The prevalence of PsA in psoriasis patients ranges from 5% to 40% [2]. However, limited research has focused on determining the incidence of *de novo* PsA or identifying predictors of disease development in psoriasis patients [3–7]. The cumulative incidence of PsA in psoriasis patients varies widely, ranging from 3.1% at 10 years [3] to 20.5% at 30 [4], and from 0.23 to 2.7 per 100 patient-years [3–9].

Some studies reported a reduction in the development of PsA in psoriatic patients treated with biologics [10–12]. However, conflicting results arose from two large-scale studies [13, 14]. A recent study showed a higher rate of onset of PsA with TNF-alpha inhibitors (TNFi) vs the other biologic classes, but a non-biological therapy reference group was not included [15]. Notably, these investigations included patients on different biologics and were susceptible to biases, such as confounding by indication, protopathic bias and ascertainment bias [16–19]. Additionally, some studies had relatively short follow-up periods [11, 14, 15]. Some studies employed topically treated patients as a control group, likely representing individuals with mild or moderate psoriasis rather than severe cases. Moreover, while most studies utilized propensity score (PS) matching to balance treatment groups and minimize biases, the potential impact of substantial unmeasured confounding variables—such as BMI, psoriasis activity, duration and psoriasis, undiagnosed PsA, psoriasis topography data, such as nail, scalp or inverse psoriasis—all could have undermined the robustness of prior reported analyses.

This longitudinal cohort study sought to compare the 10-year cumulative incidence of PsA in two groups of patients with severe psoriasis, a known risk factor for PsA: one group was treated with TNFi, while the other received narrow-band ultraviolet B (nbUVB) phototherapy. To address the limitations of prior studies, we undertook PS matching analysis of a cohort of patients on TNFi, the longest available agents, vs nbUVB therapy.

## Materials and methods

All participants gave their informed consent to participate in the study. The study protocol was reviewed and approved by the institutional ethical committee (protocol 4682/AO/19 dated 14 February 2019).

All consecutive adult patients presenting at the participating centres (Bologna, Modena, Napoli, Padova, Rome) who were prescribed TNFi treatment (etanercept, infliximab and adalimumab) or nbUVB therapy for chronic plaque psoriasis between September 2005 and September 2010 were considered for this study. Therapy interruptions lasting for <6 months between two consecutive cycles were allowed. Patients receiving combination therapies or off-label dosages were excluded. Patients were also excluded if they had PsA, as determined by a rheumatologist at baseline assessment, or if they had a history of PsA. Patients were classified as having PsA if they fulfilled the CASPAR [7]. Patients who discontinued their assigned therapy were censored at the time of treatment interruption.

Patient data including a family history of PsA, diabetes, hypertension, smoking status (current, past, never), BMI, nail psoriasis, scalp psoriasis, inverse psoriasis, Psoriasis Area Severity Index (PASI) were collected at baseline. Arthralgia, defined as any joint pain without current or past inflammatory PsA signs, and insufficient to meet the CASPAR criteria for a PsA diagnosis, was prospectively assessed at baseline and during longitudinal follow-up through routine clinical evaluations. In psoriasis patients under follow-up, those with suspected PsA—characterized by joint swelling (synovitis or dactylitis), spondylitis or enthesitis—were evaluated by a rheumatologist, who confirmed the diagnosis based on clinical, laboratory and imaging data. Clinical manifestations including tender and swollen joint counts, the presence of spondylitis/sacroiliitis, the presence and number of dactylitic digits, the presence of enthesitis, laboratory data such as the presence of RF and CRP were recorded. Plain radiographs, ultrasonography and/or MRI were performed only when clinically indicated, based on rheumatologist assessment, and not systematically at baseline or at predefined intervals during the study period.

### Statistical analysis

Results are reported as means, medians and standard deviations (SDs). Only patients with complete data for key variables were included. Group differences were assessed using Pearson’s χ^2^ test (or Fisher’s exact test for small samples) for categorical variables, and Student’s *t* test for continuous variables. The risk period spanned from cohort entry to PsA diagnosis. PsA incidence rates (per 100 person-years) with 95% CIs were calculated for both the TNFi and nbUVB groups using Poisson limits, and incidence rate ratios (IRRs) with 95% CIs were also estimated.

To address potential indication bias, PSs were calculated using multivariable logistic regression adjusting for all covariates. A 1:1 PS-matched cohort was created via nearest neighbour matching with a calliper of 0.2 [20]; unmatched patients were excluded. Standardized differences were used to compare covariate distributions between the groups, and match quality was assessed visually with density plots of pre- and post-matched PSs. Kaplan–Meier analysis, log-rank tests and Cox models were used before and after matching to evaluate PsA incidence and significant predictors over time.

Sensitivity analyses were performed by stratifying the cohort according to the key risk factor that remained slightly imbalanced after PS matching, namely the PASI score (stratified into tertiles: low, medium and high). The last-observation-carried-forward strategy was used to analyse missing covariates data during the follow-up period. All variables with a *P*-value <0.10 at univariate analysis were considered for inclusion in the multivariate analysis. We also forced all clinically relevant and imbalanced variables in a fully adjusted multivariable Cox regression model.

The proportional hazards assumption for Cox models was tested using Schoenfeld residuals (globally and per covariate), with *P* < 0.05 indicating violation (time-dependent effects). Hazard ratios with 95% CIs and *P*-values were reported. Potential effect modification was explored by testing interactions (*P* < 0.05) in the multivariate Cox model between treatment (TNFi vs nbUVB) and key baseline predictors (PASI, arthralgia, nail psoriasis, PsA family history), with interaction terms added individually to the main effects model. Analyses were conducted using IBM SPSS Statistics for Windows, version 8 (IBM Corp., Armonk, N.Y., USA) and R Statistical Software (v4.5.0; R Core Team 2024). All authors had full data access and approved the final article.

## Results

### Demographic and clinical characteristics

A total of 946 patients (497 treated with TNFi and 449 with nbUVB phototherapy) were enrolled. Two hundred forty-eight patients were treated with only one TNFi, 165 with two and 84 with three. The TNFi cohort had a total of 4752.3 person-years of follow-up, with a mean of 9.6 ± 2.6 years per person and the nbUVB cohort had 4222.1 person-years of follow-up, with a mean of 9.4 ± 5.9 years per person. Table 1 shows the baseline characteristics of the TNFi and nbUVB cohort groups before and after PS matching. Prior to matching, patients receiving TNFi were younger (mean age 50.6 vs 53.8 years) and had a higher severity of psoriasis (mean PASI 16.3 vs 11.3). Additionally, arthralgia was more common in this group (23.3% vs 18%). However, they exhibited a lower prevalence of nail psoriasis (27.8% vs 34%) and a lower family history of PsA (5.4% vs 9.8%). Furthermore, they were more likely to be smokers (37.2% vs 26.1%) and affected by dyslipidaemia (37.7% vs 28.5%), but less likely to have hypertension (30% vs 36.7%), compared with the nbUVB group. The absolute standardized differences were >0.1 for age, PASI, presence of arthralgia, presence of nail psoriasis, scalp psoriasis, family history of PsA, hypertension and dyslipidaemia (Table 1).

**Table 1.** Characteristics of the two patient cohorts before and after PS matching

	Before PS matching				After PS matching			
Variable	TNF-alpha inhibitors (*n* = 497)	Nb-UVB (*n* = 449)	*P*-value	Standardized mean difference	TNF-alpha inhibitors (*n* = 297)	Nb-UVB (*n* = 297)	*P*-value	Standardized mean difference
Age, years	50.6 ± 13.6	53.8 ± 13.9	**<0.001**	−0.224	52.2 ± 14.1	51.5 ± 13.7	0.546	0.050
Male gender, %	332 (66.8)	307 (68.4)	0.606	−0.034	195 (65.7)	192 (64.6)	0.796	−0.021
BMI, kg/m^2^	26.6 ± 4.6	26.5 ± 4.5	0.346	0.026	26.4 ± 4.5	26.3 ± 4.8	0.776	0.023
Duration of psoriasis, years	25.7 ± 10.5	25.8 ± 11.3	0.442	−0.010	26.9 ± 10.8	26.4 ± 11.2	0.625	0.040
Baseline PASI	16.3 ± 6.7	11.3 ± 6.8	**<0.001**	0.734	14.7 ± 6.5	13.6 ± 7.1	**0.039**	0.170
Presence of arthralgia, %	116 (23.3)	81 (18.0)	**0.045**	−0,131	55 (18.5)	46 (15.5)	0.326	−0,081
Presence of nail psoriasis, %	169 (34)	125 (27.8)	**0.041**	0.134	91 (30.6)	91 (30.6)	1.000	0.000
Presence of scalp psoriasis, %	313 (63)	258 (57.5)	0.083	0.113	174 (58.6)	169 (56.9)	0.678	−0.034
Presence of intergluteal/perianal psoriasis, %	167 (33.6)	152 (33.9)	0.935	−0.005	96 (32.3)	93 (31.3)	0.792	0.022
Familial history of PsA, %	27 (5.4)	44 (9.8)	**0.011**	−0.165	22 (7.4)	14 (4.7)	0.169	0.113
Smoking status, %								
Current smoker	185 (37.2)	117 (26.1)			23 (7.7)	38 (12.8)		
Past smoker	42 (8.5)	73 (16.3)			111 (37.4)	79 (26.6)		
Never	270 (54.3)	259 (57.7)	**<0.001**	−0.063	163 (54.9)	180 (60.6)	**0.007**	0.010
Presence of hypertension, %	149 (30)	165 (36.7)	**0.027**	−0.144	99 (33.3)	93 (31.3)	0.599	0.043
Presence of dyslipidaemia, %	187 (37.6)	128 (28.5)	**0.003**	0.195	93 (31.3)	95 (32)	0.860	−0.014
Presence of diabetes, %	69 (13.9)	58 (12.9)	0.664	0.028	33 (11.1)	39 (13.1)	0.451	−0.062

Statistically significant (p < 0.05) results are depicted in bold.

Values are reported as *n* (%), mean (S.D.).

Abbreviations: PASI, psoriasis area severity index.

After PS matching, 297 patients were included in each group (Table 1). The standardized differences of all potential confounders except for baseline PASI were <0.1, which was indicative of a balance match. The PS-matched TNFi cohort had a total of 2705.5 person-years of follow-up, with a mean of 9.1 ± 2.9 years per person; the PS-matched nbUVB cohort had 2654.1 person-years of follow-up, with a mean of 8.9 ± 5.4 years per person.

### Incidence of PsA in the TNFi and the nbUVB-treated groups

**Figure 1.**
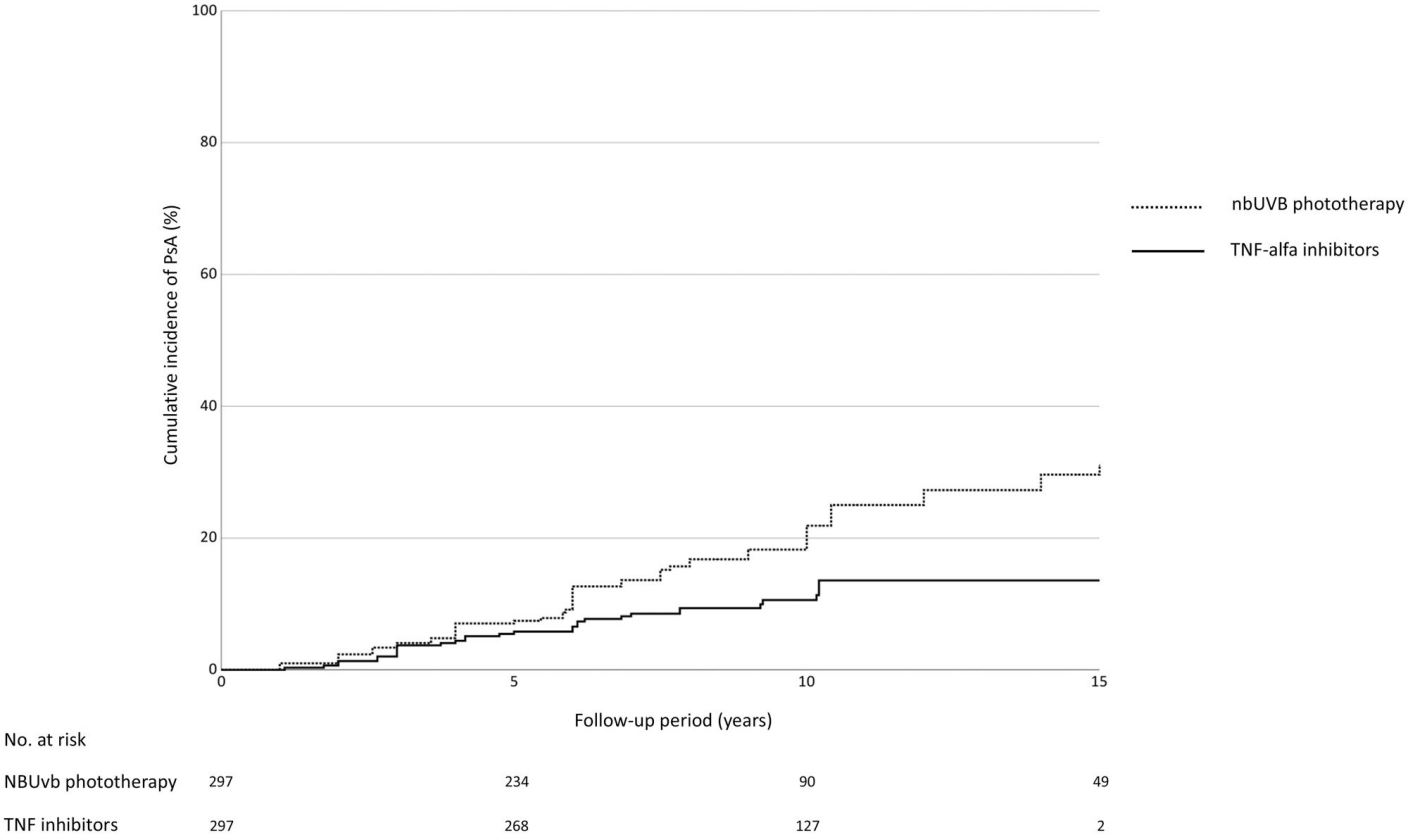
Cumulative incidence of PsA in patients treated with TNF inhibitors and with nbUVB therapy, after PS matching (*P* = 0.03, log-rank test). nbUVB, narrow-band UVB therapy

Overall, a total of 98 (16.5%) patients developed PsA: 32 (10.7%) in the cohort treated with TNFi and 66 (22.2%) in the nbUVB phototherapy-treated group (Table 2). The incidence rate of PsA cases per 100 psoriasis patients was 1.83 (95% CI: 1.632–2.028) in the whole population and 1.18 (0.84–1.52) and 2.48 (2.24–2.72) in the TNFi and nbUVB cohorts, respectively. The IRR was 2.1 (1.37–2.98, *P*: 0.0002). The cumulative incidence of PsA was 5.5 and 7.1 at 5 years and 10.6 and 18.3 at 10 years in the TNFi and nbUVB cohorts, respectively (Fig. 1) (*P* = 0.03 with log-rank test). The results are consistent with those observed before PS matching: the respective values at 5 years are 5.1 and 7.4, while at 10 years, they are 9.2 and 20.5 for the TNFi and nbUVB cohorts, respectively (*P* < 0.001 with log-rank test).

**Table 2.** Characteristics of the PS-matched population with incident PsA in the two cohorts of patients (TNF-alpha inhibitors or narrow-band ultraviolet B)

Variable	TNF-alpha inhibitors (*n* = 297)			Nb-UVB (*n* = 297)		
	PsA patients (*n* = 32)	Not PsA patients (*n* = 265)	*P*-value	PsA patients (*n* = 66)	Not PsA patients (*n* = 231)	*P*-value
Age, years	49.1 ± 10.1	52.5 ± 14.5	0.093	52.9.3 ± 10.6	51.1 ± 14.5	0.098
Male gender, %	18 (56.3)	174 (65.7)	0.293	52 (78.8)	143 (61.9)	**0.011**
BMI, kg/m^2^	28.6 ± 4.9	26.1 ± 4.3	**0.001**	26.9 ± 4.3	26.1 ± 4.9	0.941
Duration of psoriasis, years	27.4 ± 10.6	26.8 ± 10.8	0.779	27.5 ± 10.2	26.1 ± 11.4	0.540
Baseline PASI	17.7 ± 6.6	14.4 ± 6.4	**0.003**	15.8 ± 8.5	12.9 ± 6.5	**<0.001**
Presence of arthralgia, %	24 (75.0)	23 (8.7)	**<0.001**	30 (45.5)	19 (8.2)	**<0.001**
Presence of nail psoriasis, %	18 (56.3)	73 (27.5)	**<0.001**	35 (53)	56 (24.2)	**<0.001**
Presence of scalp psoriasis, %	26 (81.3)	143 (54)	**0.003**	46 (69.7)	128 (55.4)	**0.038**
Presence of intergluteal/perianal psoriasis, %	12 (37.5)	84 (31.7)	0.507	26 (39.4)	67 (29)	0.108
Familial history of PsA, %	4 (12.5)	18 (6.8)	0.244	7 (10.6)	7 (3)	**0.018**
Smoking status, %			0.944			0.978
Current smoker	12 (37.5)	99 (37.4)		18 (27.3)	61 (26.4)	
Past smoker	2 (6.3)	21 (7.9)		8 (12.1)	30 (13)	
Never	18 (56.3)	145 (54.7)		40 (60.6)	140 (60.6)	
Presence of hypertension, %	13 (40.6)	86 (32.5)	0.354	18 (27.3)	75 (32.5)	0.422
Presence of dyslipidaemia, %	13 (40.6)	80 (30.2)	0.229	24 (36.4)	71 (30.7)	0.387
Presence of diabetes, %	5 (15.6)	28 (10.6)	0.390	9 (13.6)	30 (13)	0.890
Number of painful joints	5.7 ± 3.5			4.7 ± 2.1		0.154
Number of swollen joints	3.2 ± 3.2			2.5 ± 2.3		0.317
Pattern of joint involvement, %						**0.003**
Peripheral only	27 (84.4)			32 (48.5)		
Axial only	0(0)			10 (15.1)		
Peripheral + axial	5 (15.6)			24 (36.4)		
Enthesitis	19 (59.4)			46 (69.7)		0.311
Dactylitis	18 (56.3)			39 (59.1)		0.789

Statistically significant (*P* < 0.05) results are depicted in bold.

Values are reported as *n* (%), mean (S.D.).

Abbreviations: PASI, psoriasis area severity index.

Patients developing PsA in both the groups had higher baseline PASI, more arthralgia and increased nail/scalp psoriasis (Table 2); additionally, those in the TNFi group had greater BMI, while in the nbUVB group, they were more often male. Axial PsA was less common in TNFi than nbUVB patients (15.6% vs 51.5%, *P* = 0.003). No group difference was found in enthesitis or dactylitis development.

### Predictors of PsA

In univariate analysis (adjusted for age at PsA onset, stratified by sex, with psoriasis duration as left-truncation time), several factors were significantly linked to increased PsA risk: higher BMI (HR 1.067 per unit, *P* = 0.001), PASI score (HR 1.037 per unit, *P* = 0.002), arthralgia (HR 8.988, *P* < 0.001), nail psoriasis (HR 2.891, *P* < 0.0001), scalp psoriasis (HR 2.046, *P* = 0.002), family history of PsA (HR 2.284, *P* = 0.027) and nbUVB treatment (HR 1.904, *P* = 0.004) (Table 3). Compared with BMI < 25 kg/m^2^, RRs for PsA were 1.51 (CI 0.94–2.43) for BMI 25–29.9, 1.62 (CI 0.92–2.87) for BMI 30–34.9 and 2.78 (CI 1.21–6.37) for BMI >35 kg/m^2^. In multivariable Cox regression, arthralgia (HR 9.45, *P* < 0.001), nail psoriasis (HR 1.59, *P* = 0.045), family history (HR 3.71, *P* < 0.001) and PASI score (HR 1.03 per unit, *P* = 0.009) remained independent risk factors. Conversely, TNF inhibitors were linked to a significantly reduced PsA risk (HR 0.046, *P* < 0.001) (Table 3).

**Table 3.** Variables associated with the development of PsA assessed through univariate and multivariate Cox regression

Variables	Univariate analysis HR (95% CI)	Multivariate analysis HR (95% CI)
Age, per year	0.996 (0.982–1.010)	
Male gender	1.308 (0.841–2.030)	
BMI, kg/m^2^, per unit	**1.067 (1.025–1.110)**	
Duration of psoriasis, per year	0.999 (0.980–1.017)	
Baseline PASI, per unit	**1.037 (1.013–1.062)**	**1.034 (1.009–1.061)**
Presence of arthralgia	**8.988 (5.968–13.537)**	**9.451 (6.041–14.785)**
Presence of nail psoriasis	**2.891 (1.937–4.315)**	**1.585 (1.010–2.488)**
Presence of scalp psoriasis	**2.046 (1.304–3.210)**	
Presence of intergluteal/genital psoriasis	1.354 (0.895–2.047)	
Family history of PsA	**2.284 (1.184–4.405)**	**3.706 (1.860–7.384)**
Smoking status		
Never	1	
Past	0.891 (0.572–1.388)	
Current	1.095 (0.558–2.149)	
Presence of hypertension	0.891 (0.581–1.367)	
Presence of dyslipidaemia	1.168 (0.773–1.767)	
Presence of diabetes	0.847 (0.476–1.508)	
TNF-alfa inhibitors therapy (*vs* nbUVB phototherapy)	**0.525 (0.337–0.816)**	**0.046 (0.293–0.728)**

Statistically significant (*P* < 0.05) results are depicted in bold.

Abbreviations: PASI, psoriasis area severity index.

To assess treatment effect consistency across subgroups, interactions between treatment (TNFi vs nbUVB) and key baseline predictors were examined in the Cox model. A significant interaction with nail psoriasis (*P* = 0.047) indicated TNFi’s protective effect was less pronounced in patients with baseline nail psoriasis (HR 0.47 with vs 0.23 without). A trend towards significance was also seen for arthralgia (*P* = 0.066), suggesting a similarly reduced effect (HR 0.44 with arthralgia vs 0.21 without). No significant interactions were found for other variables.

Multivariable Cox regression models stratified by baseline PASI tertiles (low, medium, high) showed that TNF inhibitors significantly reduced the risk of developing PsA across all the subgroups. Compared with phototherapy, TNF inhibitors had hazard ratios of 0.36 (low PASI), 0.11 (medium PASI) and 0.26 (high PASI), indicating a consistent protective effect, strongest in the medium PASI group. We also performed a fully adjusted multivariable Cox regression model including all clinically relevant variables, which yielded similar results, confirming the robustness of our findings. Testing with Schoenfeld residuals revealed a violation of the global proportional hazards assumption (*P* = 0.0039). Among individual covariates, family history of PsA showed a significant time-dependent effect (*P* = 0.0077), while all other covariates, including treatment group, satisfied the proportional hazards assumption (*P* > 0.05). In the final multivariable Cox regression model accounting for time-dependent effects, TNF inhibitor treatment remained associated with a significantly reduced risk of PsA compared with phototherapy (HR = 0.32, 95% CI: 0.22–0.47, *P* < 0.0001). Arthralgia (HR = 7.68, 95% CI: 5.26–11.20, *P* < 0.0001), nail psoriasis (HR = 1.93, 95% CI: 1.34–2.78, *P* = 0.0004) and higher baseline PASI score (HR = 1.03 per point increase, 95% CI: 1.01–1.05, *P* = 0.0096) were independently associated with increased PsA risk. No other variables showed statistically significant associations. The final model demonstrated good discriminative ability, with a concordance index (*C*-index) of 0.802 (standard error = 0.02).

## Discussion

Incomplete and contradictory data exist on PsA incidence and associated risk factors in psoriasis patients under biologic therapy [10–14, 19]. Some studies suggest a decreased risk of PsA in psoriasis patients treated with biologics compared with those receiving phototherapy, topical therapy or conventional synthetic disease-modifying antirheumatic drugs [10–12]. However, some studies have suggested an increased risk of PsA associated with TNFi use. Among these, three claim-data-based studies reported such an association when comparing TNFi, respectively, with non-systemic and non-biologic systemic therapies [13], with oral therapies or phototherapy [14] and with IL-17/IL-23 inhibitors [19]. Similarly, a retrospective observational study found an increased risk when TNFi were compared with patients treated with IL-17/IL-23 inhibitors [15].

Collectively, these studies were limited due to differences in patient characteristics in study groups, several biases and the relatively short follow-up periods [15–18]. Our study demonstrates that continuous, long-term treatment approaching a decade with TNFi significantly decreases the incidence of PsA in severe psoriasis patients compared with long-term nbUVB phototherapy.

The use of PS matching effectively addressed baseline differences between the two groups of patients, minimizing potential confounding factors in the analysis of PsA development. While PS matching is a valuable method for adjusting confounding variables and reducing treatment selection bias, it cannot entirely replace a randomized trial. Previous retrospective studies using electronic records may have used PS matching, but they often failed to account for unmeasured factors influencing PsA development, such as PASI scores, arthralgia, nail psoriasis, BMI and family history [11, 12–15, 19]. Considering these limitations, the interpretation of the existing evidence must be cautious. In our study, we employed a comprehensive approach by integrating detailed baseline patient profiles and clinical characteristics to strengthen the validity of comparisons between two psoriasis treatment modalities over nearly a decade. Additionally, all patients received longitudinal rheumatologist assessments to ensure accurate diagnosis timing. We also accounted for arthralgia, which was significantly more prevalent in the TNFi-treated group, creating an imbalance between cohorts that was corrected after PS matching. This strategy aimed to mitigate protopathic bias, a potential confounding factor that may have influenced analyses in earlier studies [13–15, 19].

Matching yielded well-balanced TNFi and nbUVB cohorts with minor baseline PASI differences. Although psoriasis activity is linked to increased PsA risk [4, 5, 21], the marginal difference in psoriasis severity between the groups (mean PASI 14.7 ± 6.5 vs 13.6 ± 7.1) is unlikely to have substantially affected the analysis.

Multivariate Cox regression confirmed an independent association between the use of TNFi and a substantially lower incidence of PsA in psoriasis patients, indicating less than half the risk of developing PsA compared with those treated with phototherapy. Most prior studies have focused on cross-sectional analyses to determine PsA point prevalence, with few longitudinal investigations into *de novo* PsA incidence [22]. In our study, overall PsA incidence was 1.83 per 100 psoriasis patients, aligning with previous rates of 1.37–1.55 [10–12]. Eder *et al.*’s prospective study [5], focused on phototherapy centres, reported a similar PsA incidence (2.7 vs 2.48 cases per 100 psoriasis patients) to our nbUVB phototherapy group. The lower rate of new PsA observed in patients treated with TNFi compared with those receiving phototherapy may be attributed to several factors. The decreased activity of TNF, modulated by TNFi, in the joints and entheses may have aborted early pathogenic steps of PsA impairing the interactions between the cells involved in disease development. The central role of TNF in PsA immunopathogenesis is well established, with its presence in inflamed synovium and entheses activating immune cells, fibroblasts and osteoclasts, which then release cytokines, chemokines and alarmins, amplifying inflammation and tissue damage [23, 24]. Phototherapy was chosen as the comparator because it controls skin psoriasis without systemic immunosuppressive effects. This allowed us to assess whether systemic immune modulation, rather than skin clearance alone, is necessary to prevent PsA. Our findings suggest that systemic immunoregulation, as achieved with TNFi therapy, may be critical in reducing PsA risk. Additionally, the protective role of prolonged TNFi therapy on the development of PsA could be attributed to more effective control of psoriasis remission that is supported by the recognized association between psoriasis disease activity and PsA risk [4, 5, 21].

Besides describing the lower cumulative incidence of *de novo* PsA in patients treated with TNFi compared with those treated with nbUVB, our study confirmed some already reported clinical predictors of PsA that have been noted over an extended period in psoriasis patients including nail psoriasis [4, 5, 25] that may be related to the nail as part of the ‘enthesis organ’ [26]. Interestingly, we also found a significant interaction between treatment and nail psoriasis (*P* = 0.047). This suggests that while TNFi therapy reduced the overall incidence of PsA, its protective effect relative to nbUVB was less marked in patients with nail involvement (HR for TNFi vs nbUVB: 0.47) compared with those without (HR: 0.23). This finding highlights that patients with nail psoriasis, a strong risk factor for PsA, still benefit from TNFi in terms of PsA prevention compared with phototherapy, but the magnitude of this relative benefit is reduced.

We also confirmed the previously reported association between greater psoriasis activity and an increased risk of PsA development as reported in multiple studies [4, 5, 13, 21, 27]. Accordingly, psoriasis severity is recognized as one of the four major long-term risk factors for PsA development in the EULAR points to consider on pre-PsA [28].

Moreover, our study underscores arthralgia’s pivotal role as a strong predictor of PsA development in psoriasis patients (a 9-fold increased risk in multivariate analysis), consistent with prior findings of arthralgia as a preclinical symptom of disease progression [29, 30]. Before PS matching, TNFi patients had a higher arthralgia prevalence than nbUVB patients. This suggests potential protopathic bias in dermatologic settings, where clinicians might prescribe PsA-effective therapies for psoriasis patients with musculoskeletal symptoms [15–19]. This selection could create a spurious association between PsA-effective therapies and increased PsA incidence, especially in electronic health database studies. Indeed, many retrospective studies lack detailed records of early PsA indicators like arthralgia, making it difficult to ascertain if PsA developed post-treatment or was already subclinically progressing [13–15, 19]. Our interaction analysis also showed a trend (*P* = 0.066) suggesting arthralgia might modify the TNFi vs nbUVB treatment effect. Specifically, TNFi’s protective effect, while still evident, appeared somewhat attenuated in patients with baseline arthralgia (HR for TNFi vs nbUVB 0.44) compared with those without (HR 0.21). This observation, coupled with arthralgia’s strong predictive capacity for PsA, suggests that while TNFi are beneficial, the journey from non-specific musculoskeletal symptoms to diagnosed PsA in patients on TNFi warrants further investigation.

Finally, in our initial multivariable analysis, a family history of PsA was an independent predictor of future PsA development. However, when time-dependent effects were modelled, this association was no longer significant, suggesting that the influence of family history on PsA risk may not be constant over time. Cross-sectional data showed that first-degree relatives (FDRs) of PsA patients have a significantly higher risk (*λ* = 14–55) compared with the general population [31–33]. While most evidence is cross-sectional rather than longitudinal, it consistently confirms an elevated PsA risk in psoriasis patients with affected FDRs. Reflecting these observations, two international consensus statements have underscored the importance of family history—particularly having an FDR with PsA—as a key risk factor for PsA development, despite the limited evidence [28, 34]. Further prospective studies are needed to better clarify the dynamic role of familial predisposition in PsA onset.

Our study has some limitations. Despite employing PS matching to create a balanced cohort resembling the conditions of an RCT within a non-interventional study setting, we recognize the possibility of unmeasured confounders (e.g. socioeconomic status, education level, depression) influencing the outcomes. Additionally, the generalizability of the study may be limited as all the participants were recruited from psoriasis referral centres, possibly leading to an over-representation of more severe psoriasis forms and, possibly, patients with longer duration of psoriasis. Although TNF inhibitors were the primary biologics available at the time of cohort recruitment (2005–2010), and newer classes such as IL-17 and IL-23 inhibitors have since gained popularity, TNF inhibitors continue to be widely used across many European countries due to the availability of biosimilars and associated pharmaco-economic considerations. It should be noted that certolizumab and golimumab were not included in this study; therefore, caution is warranted in generalizing our findings to the entire class of TNF inhibitors.

Furthermore, although prospective assessment of arthralgia (at baseline and follow-up through routine clinical evaluations) minimized recall bias, some patients with arthralgia could still have had subclinical PsA not meeting CASPAR criteria. This limitation, common in longitudinal studies of PsA progression, underscores the difficulty in identifying true preclinical disease stages.

In conclusion, this prospective cohort study found that prolonged TNFi treatment plays a protective role in PsA development in severe psoriasis patients. Further prospective studies assessing the impact of other biologic treatments on *de novo* PsA development in psoriatic patients will be able to provide more insight into these mechanisms and whether IL-23/17 axis blockers are even better at PsA prevention [15, 19]. Our findings additionally confirmed key clinical predictors of *de novo* PsA, including nail psoriasis, family history of PsA, higher PASI scores and arthralgia. A multidisciplinary approach involving dermatologists and rheumatologists in identifying and managing arthralgia could be crucial in modifying disease progression. Future research should investigate the mechanisms linking arthralgia to PsA and assess whether early interventions can prevent long-term joint damage. These findings highlight the essential role of dermatologists in identifying psoriasis patients at increased PsA risk [28].

## Data Availability

The data that support the findings of this study are available from the corresponding author upon reasonable request.

## References

[1] Michalek IM , Loring B, John SM. A systematic review of worldwide epidemiology of psoriasis. J Eur Acad Dermatol Venereol 2017;31:205–12. 27573025 10.1111/jdv.13854

[2] Ritchlin CT , Colbert RA, Gladman DD. Psoriatic arthritis. N Engl J Med 2017;376:957–70. 10.1056/NEJMra1505557 28273019

[3] Wilson FC , Icen M, Crowson CS et al Incidence and clinical predictors of psoriatic arthritis in patients with psoriasis: a population-based study. Arthritis Rheum 2009;61:233–9. 19177544 10.1002/art.24172 PMC3061343

[4] Christophers E , Barker JN, Griffiths CE et al The risk of psoriatic arthritis remains constant following initial diagnosis of psoriasis among patients seen in European dermatology clinics. J Eur Acad Dermatol Venereol 2010;24:548–54. 19874432 10.1111/j.1468-3083.2009.03463.x

[5] Eder L , Haddad A, Rosen CF et al The incidence and risk factors for psoriatic arthritis in patient with psoriasis– a prospective cohort study. Arthritis Rheumatol 2016;68:915–23. 10.1002/art.39494 26555117

[6] Zabotti A , De Lucia O, Sakellariou G et al Predictors, risk factors, and incidence rates of psoriatic arthritis development in psoriasis patients: a systematic literature review and meta-analysis. Rheumatol Ther 2021;8:1519–34. 10.1007/s40744-021-00378-w PMC8572278 34596875

[7] Taylor W , Gladman D, Helliwell P et al; CASPAR Study Group. Classification criteria for psoriatic arthritis: development of new criteria from a large international study. Arthritis Rheum 2006;54:2665–73. 16871531 10.1002/art.21972

[8] Nguyen U-SD , Zhang Y, Lu N et al The smoking paradox in the development of psoriatic arthritis among psoriasis patients—a population-based study. Ann Rheum Dis 2018;77:119–23. 29102956 10.1136/annrheumdis-2017-211625 PMC5978759

[9] Lindberg I , Lilja M, Geale K et al Incidence of psoriatic arthritis in patients with skin psoriasis and associated risk factors: a retrospective population-based cohort study in Swedish routine clinical care. Acta Derm Venereol 2020;100:adv00324. 10.2340/00015555-3682 PMC9309831 33135771

[10] Gisondi P , Bellinato F, Targher G, Idolazzi L, Girolomoni G. Biological disease-modifying antirheumatic drugs may mitigate the risk of psoriatic arthritis in patients with chronic plaque psoriasis. Ann Rheum Dis 2022;81:68–73. 10.1136/annrheumdis-2021-219961 34144965

[11] Acosta Felquer ML , LoGiudice L, Galimberti ML et al Treating the skin with biologics in patients with psoriasis decreases the incidence of psoriatic arthritis. Ann Rheum Dis 2022;81:74–9. 34281904 10.1136/annrheumdis-2021-220865

[12] Shalev Rosenthal Y , Schwartz N, Sagy I, Pavlovsky L. Psoriatic arthritis incidence among patients receiving biologic medications for psoriasis: a nested case control study. Arthritis Rheumatol 2021;1:845. 10.1002/art.41946 34423909

[13] Merola JF , Tian H, Patil D et al Incidence and prevalence of psoriatic arthritis in patients with psoriasis stratified by psoriasis disease severity: retrospective analysis of an electronic health records database in the United States. J Am Acad Dermatol 2022;86:748–57. 34547358 10.1016/j.jaad.2021.09.019

[14] Meer E , Merola JF, Fitzsimmons R et al Does biologic therapy impact the development of PsA among patients with psoriasis? Ann Rheum Dis 2022;81:80–6. 10.1136/annrheumdis-2021-220761 34615637

[15] Gisondi P , Bellinato F, Galeone C et al Risk of developing psoriatic arthritis in patients with psoriasis initiating treatment with different classes of biologics. Ann Rheum Dis 2025;84:435–41. 39919973 10.1016/j.ard.2025.01.006

[16] Koehm M , Behrens F. Association between biological immunotherapy for psoriasis and time to incident inflammatory arthritis: limitations and opportunities. RMD Open 2023;9:e003166. 37734874 10.1136/rmdopen-2023-003166 PMC10514622

[17] Ogdie A , Scher JU. Prevention of psoriatic arthritis: the next frontier. Lancet Rheumatol 2023;5:e170–1. 38251512 10.1016/S2665-9913(23)00055-3

[18] Soriano ER , Ogdie A. Can early aggressive treatment of psoriasis prevent psoriatic arthritis? A debate at the GRAPPA annual meeting. J Rheumatol 2023;50:8–10. 37527866 10.3899/jrheum.2023-0506

[19] Singla S , Putman M, Liew J, Gordon K. Association between biological immunotherapy for psoriasis and time to incident inflammatory arthritis: a retrospective cohort study. Lancet Rheumatol 2023;5:e200–7. 38251522 10.1016/S2665-9913(23)00034-6

[20] Austin PC. A comparison of 12 algorithms for matching on the propensity score. Stat Med 2014;33:1057–69. 24123228 10.1002/sim.6004 PMC4285163

[21] Ogdie A , Langan S, Love T et al Prevalence and treatment patterns of psoriatic arthritis in the UK. Rheumatology 2013;52:568–75. 23221331 10.1093/rheumatology/kes324 PMC3573270

[22] Karmacharya P , Chakradhar R, Ogdie A. The epidemiology of psoriatic arthritis: a literature review. Best Pract Res Clin Rheumatol 2021;35:101692. 34016528 10.1016/j.berh.2021.101692

[23] Ritchlin C , Haas-Smith SA, Hicks D et al Patterns of cytokine production in psoriatic synovium. J Rheumatol 1998;25:1544–52. 9712099

[24] Bradley JR. TNF-mediated inflammatory disease. J Pathol 2008;214:149–60. 18161752 10.1002/path.2287

[25] Langenbruch A , Radtke MA, Krensel M et al Nail involvement as a predictor of concomitant psoriatic arthritis in patients with psoriasis. Br J Dermatol 2014;171:1123–8. 25040629 10.1111/bjd.13272

[26] McGonagle D. Enthesitis: an autoinflammatory lesion linking nail and joint involvement in psoriatic disease. J Eur Acad Dermatol Venereol 2009;23:9–13. 19686380 10.1111/j.1468-3083.2009.03363.x

[27] Gelfand JM , Gladman DD, Mease PJ et al Epidemiology of psoriatic arthritis in the population of the United States. J Am Acad Dermatol 2005;53:573–7. 10.1016/j.jaad.2005.03.046 16198775

[28] Zabotti A , De Marco G, Gossec L et al EULAR points to consider for the definition of clinical and imaging features suspicious for progression from psoriasis to psoriatic arthritis. Ann Rheum Dis 2023;82:1162–70. 10.1136/ard-2023-224148 37295926

[29] Eder L , Polachek A, Rosen CF et al The development of psoriatic arthritis in patients with psoriasis is preceded by a period of nonspecific musculoskeletal symptoms: a prospective cohort study. Arthritis Rheumatol 2017;69:622–9. 10.1002/art.39973 27792862

[30] De Marco G , Zabotti A, Baraliakos X et al Characterisation of prodromal and very early psoriatic arthritis: a systematic literature review informing a EULAR taskforce. RMD Open 2023;9:e003143. 37349122 10.1136/rmdopen-2023-003143 PMC10314670

[31] Karason A , Love TJ, Gudbjornsson B. A strong heritability of psoriatic arthritis over four generations–the Reykjavik Psoriatic Arthritis Study. Rheumatology 2009;48:1424–8. 19741010 10.1093/rheumatology/kep243

[32] Chandran V , Schentag CT, Brockbank JE et al Familial aggregation of psoriatic arthritis. Ann Rheum Dis 2009;68:664–7. 10.1136/ard.2008.089367 18524791

[33] FitzGerald O , Haroon M, Giles JT, Winchester R. Concepts of pathogenesis in psoriatic arthritis: genotype determines clinical phenotype. Arthritis Res Ther 2015;17:115. 10.1186/s13075-015-0640-3 PMC4422545 25948071

[34] Perez-Chada LM , Haberman RH, Chandran V et al Consensus terminology for preclinical phases of psoriatic arthritis for use in research studies: results from a Delphi consensus study. Nat Rev Rheumatol 2021;17:238–43. 10.1038/s41584-021-00578-2 PMC7997804 33589818

